# Highly Sensitive Plasmonic Biosensors with Precise Phase Singularity Coupling on the Metastructures

**DOI:** 10.3390/bios12100866

**Published:** 2022-10-12

**Authors:** Joelle Youssef, Shaodi Zhu, Aurelian Crunteanu, Jean-Christophe Orlianges, Ho-Pui Ho, Renaud Bachelot, Shuwen Zeng

**Affiliations:** 1 Light, Nanomaterials & Nanotechnologies (L2n), CNRS-ERL 7004, Université de Technologie de Troyes, 10000 Troyes, France; 2 Department of Biomedical Engineering, The Chinese University of Hong Kong, Shatin, N.T., Hong Kong 999077, China; 3 XLIM Research Institute, UMR 7252 CNRS/University of Limoges, 123 Avenue Albert Thomas, 87060 Limoges, France

**Keywords:** surface plasmons, phase-change material, vanadium dioxide, phase detection, Goos–Hänchen shift, Goos–Hänchen sensitivity, cancer biomarkers

## Abstract

In this paper, we demonstrated the ability of a plasmonic metasensor to detect ultra-low refractive index changes (in the order of ∆n = 10^−10^ RIU), using an innovative phase-change material, vanadium dioxide (VO_2_), as the sensing layer. Different from current cumbersome plasmonic biosensing setups based on optical-phase-singularity measurement, our phase signal detection is based on the direct measurement of the phase-related lateral position shift (Goos–Hänchen) at the sensing interface. The high sensitivity (1.393 × 10^8^ μm/RIU for ∆n = 10^−10^ RIU), based on the Goos–Hänchen lateral shift of the reflected wave, becomes significant when the sensor is excited at resonance, due to the near-zero reflectivity dip, which corresponds to the absolute dark point (lower than 10^−6^). GH shifts in the order of 2.997 × 10^3^ μm were obtained using the optimal metasurface configuration. The surface plasmon resonance (SPR) curves (reflectivity, phase, GH) and electromagnetic simulations were derived using the MATLAB programming algorithm (by the transfer matrix method) and Comsol modeling (by finite element analysis), respectively. These results will provide a feasible way for the detection of cancer biomarkers.

## 1. Introduction

For nearly a decade, the world has been witnessing innovative medical advances, more recently the various COVID-19 vaccines, which tend to increase the lifespan of humanity. Consequently, an augmented percentage of people diagnosed with cancerous diseases is being recorded due to the expanding number of people in old age. Cancer is one of the leading death-causing diseases according to the World Health Organization [1]. Statistics by an international agency for research on cancer show that by 2040, cancer cases are expected to increase (to 29.5 million), along with a rise in the number of deaths (to 16.4 million) worldwide [2]. This, instantly, puts urgency on the development of early diagnosis procedures, since cancer treatments are shown to be highly effective in early stages of detection. Current diagnosis methods include laboratory tests, such as the enzyme-linked immunosorbent assay (ELISA) that detects some antibodies in blood [3], the complete blood count (CBC) test that measures the different types of blood tests (red and white cells, platelets), cytogenetic analysis that evaluates any change in the number of chromosomes in white cells, and some imaging techniques such as computerized tomography (CT) scanning and magnetic resonance imaging (MRI) [4]. These approaches are time-consuming and expensive, requiring complex equipment, and some of them cannot detect cancer in its earliest stages. MRI, for example, cannot detect cancerous cells below the size of 1 cm [5]. Accordingly, alternatives known as ‘plasmonic biosensors’ have been suggested as substitute tools.

Plasmonic biosensors are used to detect the presence of specific biomarkers (DNA, RNA, protein) in blood, serum, or urine and test the efficacy of chemotherapy treatments. They are known for their high sensibility, fast and early detection ability, accuracy, flexibility, easy access (point-of-care testing), and cheapness [5]. They rely, principally, on the excitation of ‘surface plasmon polaritons’—collective oscillations of free electrons—excited at the interface between a metal and a dielectric. Plasmons have a specific momentum, which depends on the nature of the metal–dielectric employed and the surrounding medium [6]. A resonance occurs when the momentum of incident light (defined by its wavelength and incident angle) matches that of the plasmons; in this case, nearly all of the incident energy is transferred to the surface plasmons and, as a direct result, a dark band is observed in the reflected spectrum. A microfluidic channel is, afterward, installed, through which deionized water is initially flown, followed by the sample solution; the presence of cancerous biomarkers is distinguished according to the modification in the refractive index they introduce. This change can be tracked through several schemes: change in resonance angle, minimum reflectivity, and wavelength [7,8,9,10]. These practices have shown poor performances, especially when subjected to extremely small refractive index modifications (in the order of 10^−6^) and, therefore, cannot detect cancer in its early stages (supporting information Tables S2–S5). Instead, the latest research has started considering phase measurements that take advantage of the singular behavior of the phase when excited at the resonance angle [7,8,11]. However, the detection of phase changes requires interferometry schemes with complex experimental setups [8].

A more significant order of the phase, known as the Goos–Hänchen (GH) shift, can be more pronounced and easily detected; it is a lateral shift of the reflected beam, when totally internally reflected at the boundary between two materials of distinct refractive indexes [12,13]. Theoretical approaches have been developed to characterize this shift and quantify it in terms of mathematical formulations. Two key opposing points of view are cited: conservation of energy (or the energy flux method) and the stationary phase method for a finite wave packet [14]. The second approach has been proven to yield better approximations, in comparison to the experimental models. Academic studies have reported the inclusion of additional layers to enhance the GH shift and sensitivity: perovskite [8], graphene [7,15], transition metal dichalcogenides (TMDs) such as MoS_2_ and WS_2_, hybrid structures such as silicon–graphene, and/or gold nanoparticles and metasurfaces [10,15]. These configurations have proven good efficiencies for detecting low refractive index changes ~10^−9^, as they promote an effective charge transfer and, hence, an enhanced electromagnetic field at the sensing layer. Although these materials have reported high theoretical GH sensitivities at low refractive index changes (1.5458 × 10^9^ μm/RIU for perovskite [8]), they still lag experimentally due to their non-negligible surface roughness at the nanoscale and the impossibility to achieve thickness accuracies at the angstrom scale [16]. As a direct result, the predicted theoretical values are hardly reachable. Recently, phase-change materials have started gaining popularity due to their distinctive nature, which allows them to alter the physical properties of nanostructures; this is the result of a fast modification in their structure caused by an external thermal, electrical, or optical stimulus. Physically, the change in the crystallography of the material is observed via a notable variation in the dielectric function [17].

SPR detection, based on phase-change materials, is expected to yield better experimental GH sensitivities due to their high absorption coefficients in the visible and infrared, at low thicknesses. Moreover, the dielectric function can be easily readjusted when it seems to be deviated from its optimal value, by regulating the crystallization conditions [17,18]. SPR sensors based on GeSbTe (germanium–antimony–tellurium) [19] and Sb_2_S_3_ (antimony trisulfide) [20] as phase-change materials have been already theoretically modeled but yielded poor results (GH shifts in the order of a few tens of micrometers for Sb_2_S_3_). This report concentrates on another type of phase-change material, known as vanadium dioxide (VO_2_). Experimental studies indicate high absorption coefficients in the visible–infrared region at high temperatures, so, a deep reflection and large GH shift can be anticipated. The insulator-to-metal behavior was first discovered by Morin in 1959, during their studies on the relation between temperature and electrical conductivity; then, a few years after, the same laboratory (Bell Labs) issued the first experimental datasheet describing the optical properties of VO_2_ [17]. VO_2_ undergoes a reversible structural transition from insulator (monoclinic phase) to absorbing metal (tetragonal phase), when heated at 68 °C, which is known as the metal–insulator-transition temperature (MIT). This effect has been largely exploited in optical and optoelectronic instruments such as optical data storage devices, plasmonic modulators, and hybrid tunable VO_2_ waveguides, due to their capability to retain and ‘memorize’ the state when the trigger is removed [17,18,21]. Recently, biosensors based on vanadium dioxide are starting to attract attention due to their ability to detect glucose in blood. The surface of the phase-change material was properly functionalized to detect the presence of the target molecule. Glucose oxydase (GOx) enzyme, dissolved in PBS buffer solutions (1.5 mg/L) along with 5% of glutaraldehyde solution, was injected on the VO_2_ surface for 1 min and then left to dry for 24 h. The efficiency of the immobilization of the enzyme by covalent cross-linking was, subsequently, tested as a function of glucose concentration and yielded promising results. This will pave the way for the development of highly sensitive plasmon sensors to be used for more advanced medical applications [22].

## 2. Materials and Methods

**Figure 1:**
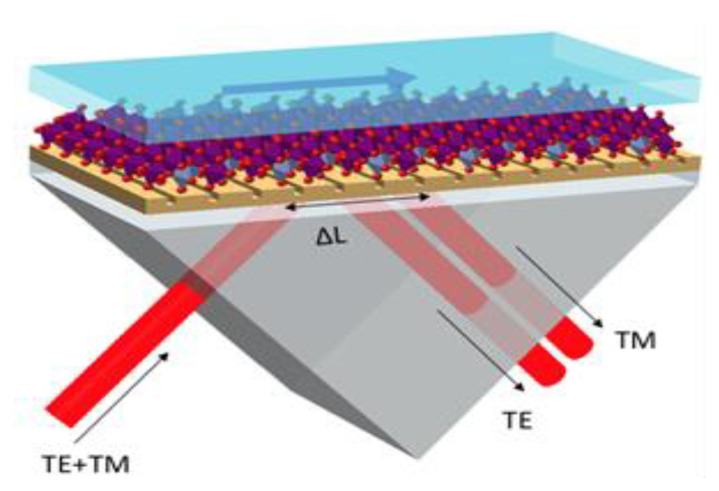
Schematic representing the Kretschmann SPR sensor based on a gold metasurface: prism, glass substrate, gold, and Vanadium dioxide as the sensing layer.

The excitation of the surface plasmon polaritons is usually performed through various methods: prism-coupled SPR, SPR waveguide, and diffraction-grating-coupling-based SPR sensing. The well-known Kretschmann configuration, based on a prism coupling of incident light into a metal-dielectric substrate, is presented here. The model depicted in Figure 1, consists of an SF11 prism, a BK7 glass substrate with a fixed thickness of 100 nm, gold film (with or without metasurface), vanadium dioxide (VO_2_) as the sensing layer, and water as a calibration/testing medium. The variables used for evaluating the performance of the sensor include minimum reflectivity, GH shift, and GH sensitivity, with respect to a refractive index change.

The dispersion relation of gold was obtained from the Lorentz–Drude model and that of VO_2_ from experimental data. The refractive index of the prism and glass substrate are obtained using equations described in supporting information. The transition temperature of VO_2_ is theoretically set at 68 °C [18,21]; thus, the two chosen temperatures represent both the insulating and conducting states of the phase-change material. Simulations were performed using MATLAB as the programming algorithm and the transfer matrix method (TMM) (Section 1: Methods and Theoretical Modeling) for obtaining the SPR curves (reflectivity, phase, GH) and COMSOL Multiphysics 6.0 for evaluating the electric field inside the structure.

## 3. Results

The GH shift is expected to increase with the addition of VO_2_ as a sensing layer due to the magnification of the electric field within the structure. In this section, the performance of the sensor is deeply analyzed according to certain parameters such as minimum reflectivity, GH shift, and sensitivity. However, beforehand, a general presentation of the physical and optical properties of this material is required.

**Figure 2:**
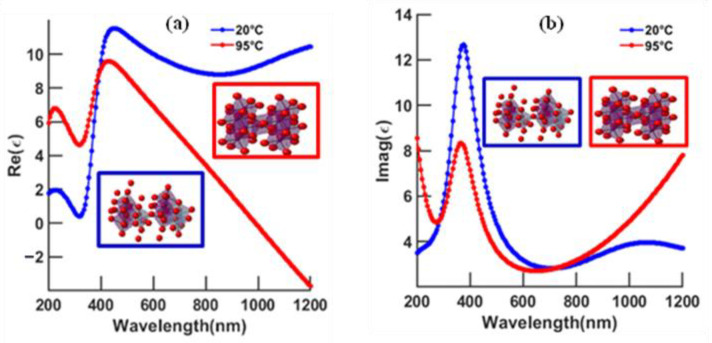
Experimental dielectric function: (**a**) real part and (**b**) imaginary part of VO_2_ at 20 °C and 95 °C.

At 95 °C (see above, the metal–insulator temperature of VO_2_ is ~68 °C [17,18]), the real component of the permittivity of VO_2_, as shown in Figure 2a, goes through a linear decrease after 400 nm and reaches negative values (0 at approximately 985 nm). On the other hand, the imaginary component, Figure 2b, in the range of 400–700 nm, experiences a reduction and then rises again for longer wavelengths. As for 20 °C, no significant modifications of either the real or the imaginary components of the phase-change material in the UV–visible–IR region is shown. The metallic behavior can then be summarized at 95 °C, for excitations approximately greater than 985 nm, which is the starting point at which the real part of the dielectric function begins to switch to negative values, and the imaginary part of the refractive index (n = $\sqrt{\varepsilon }$ = n_r_ + ki) describing the absorption of the metal increases.

### 3.1. Continuous Gold Model

**Figure 3:**
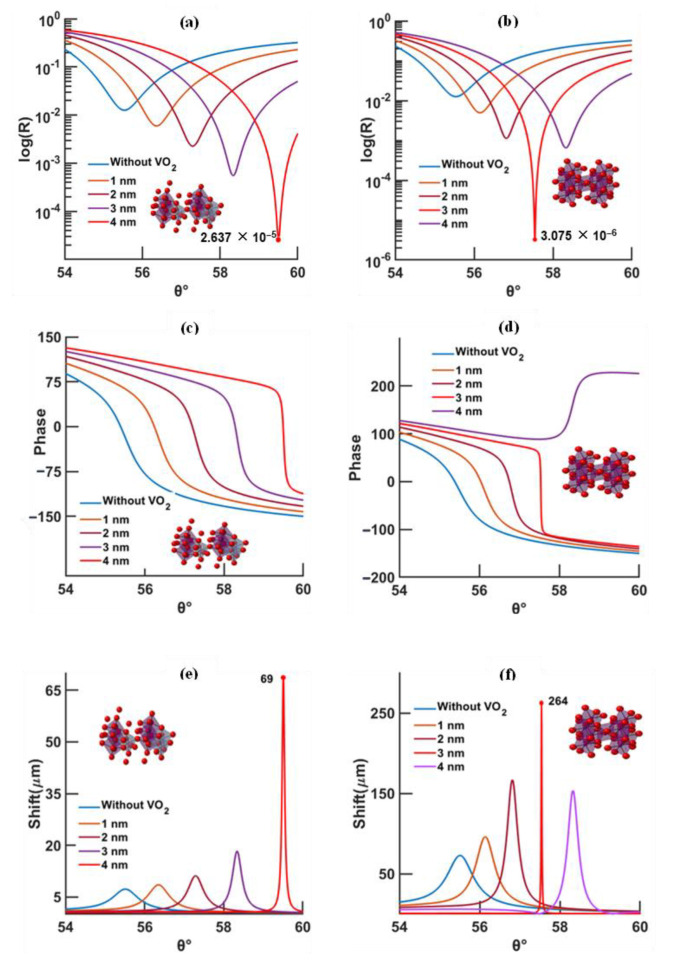
SPR curves with respect to the thickness of VO_2_ calculated by TMM model, for an excitation of 630 nm, VO_2_ temperature of 20 °C (left) and 95 °C (right), and a gold thickness of 46 nm; (**a**,**b**) reflectivity, (**c**,**d**) phase, and (**e**,**f**) Goos–Hänchen shift (absolute value).

**Figure 4:**
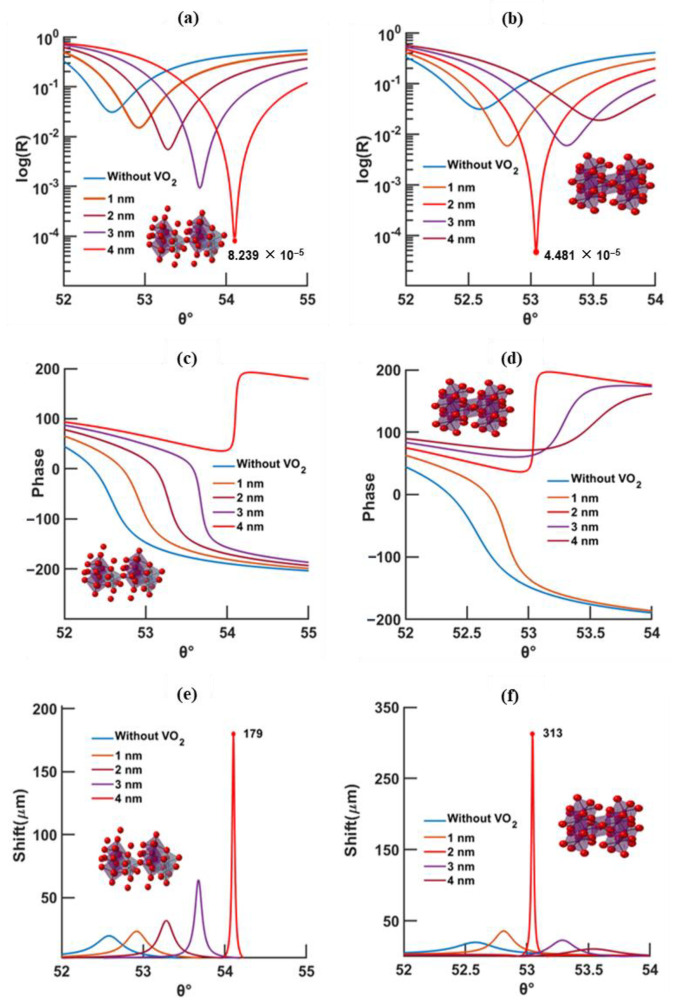
SPR curves with respect to the thickness of VO_2_ calculated by TMM model, for an excitation of 785 nm, VO_2_ temperature of 20 °C (left) and 95 °C (right), and a gold thickness of 46 nm; (**a**,**b**) reflectivity, (**c**,**d**) phase, and (**e**,**f**) Goos–Hänchen shift (absolute value).

**Figure 5:**
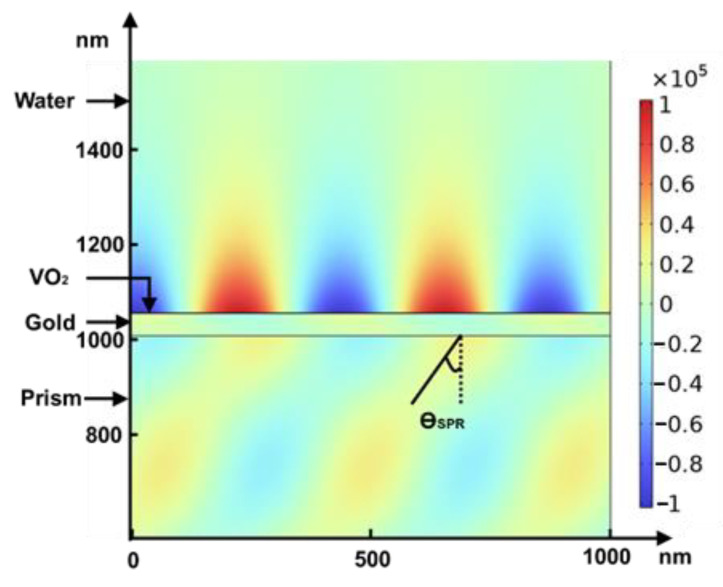
Electric field (y component in V/m) distribution inside the structure, given that the thicknesses of the gold film and VO_2_ are 47 and 1 nm, respectively, excitation is 630 nm, and temperature is 20 °C.

Taking advantage of the singular behavior displayed at the resonance angle, detection based on phase is reported here [7,8,11]. The Goos–Hänchen shift, which is related to the phase delay of the reflected wave upon resonance, is employed for evaluating the sensitivity of the SPR device [12,13]. Due to its absorbing properties, the phase-change material is responsible for lowering the reflected signal, improving the GH shift and enhancing the electric field at the sensing layer. Figure 3 and Figure 4 illustrate and compare the effects of wavelength (630 and 785 nm), temperature (20 and 95 °C), and VO_2_ thickness (0 to 4 nm) on the minimum reflectivity, phase, and GH shift at fixed gold thickness (46 nm). Furthermore, Figure 5 depicts the enhancement effect introduced at the surface of the sensing layer.

According to Figure 3a–f, for a 630 nm excitation, the lowest reflectivity of ~3.075 × 10^−6^ is set at 3 nm for a temperature of 95 °C; the maximum GH shift of ~264 μm is higher than that at 20 °C (69 μm), along with a more confined GH curve profile. Since this study is mainly based on the evaluation of the GH shift, a confined GH curve is needed for a fine angular tuning of the sensor. For a higher excitation wavelength of 785 nm, Figure 4a–f, the lowest reflectivity, at 20 and 95 °C, is comparable in value (8.239 × 10^−5^ and 4.481 × 10^−5^, respectively) but occurs at different thicknesses of the phase-change material (4 nm at 20 °C and 2 nm at 95 °C).

A decrease in the resonance angle is manifested by increased temperatures and excitation wavelengths, which may be explained by the reduction in the real part of the permittivity (Figure 3 and Figure 4) when subjected to these conditions. At the same excitation, ε_real_ at 20 °C > ε_real_ at 95 °C, and same temperature, ε_real_ at 630 nm > ε_real_ at 785 nm, this will, consequently, lead to a smaller k vector (Equation (S9) in supporting information) and, therefore, a smaller angle to excite the surface plasmon waves.

Tables S2–S5 and Equations (S13)–(S17) in supporting information demonstrate that the phase-related sensitivity yields more prominent results than the ones based on angular detection, with a—1.183 × 10^5^ °/RIU difference in sensitivity between both, for ∆n = 1.2 × 10^−6^ RIU (Equations (S15)–(S17) in supporting information and Table S2). Furthermore, the addition of the phase-change-material sensing layer significantly improved the GH shift for both wavelengths and temperatures (Tables S2 and S3), from 94.02 μm for the bare gold to 2.147 × 10^3^ μm for VO_2_ at 95 °C and excited at 630 nm, and the GH sensitivity (Tables S2 and S3), from 9.736 × 10^3^ μm/RIU for the bare gold to 7.323 × 10^6^ μm/RIU when Δn = 1.2 × 10^−6^ RIU and for VO_2_ at 95 °C excited at 630 nm. Comparing the two excitation wavelengths, the best configuration is set when the sensor is excited at 630 nm, gold thickness is 47 nm, and VO_2_ 2 nm at 95 °C: 2.147 × 10^3^ μm GH shift, 9.191 × 10^4^ μm/RIU for ∆n = 0.02 GH sensitivity, and 7.323 × 10^6^ μm/RIU for ∆n = 1.2 × 10^−6^ GH sensitivity. Contrarily, for the higher excitation wavelength 785 nm, lower temperature yielded better GH shift (7.891 × 10^2^ μm, when gold and VO_2_ thicknesses are 48 and 2 nm, respectively).

In conclusion, according to Figure 3 and Figure 4 and Tables S2–S6 in supporting information:

1. At higher temperatures and wavelengths, decreases in the resonance angles for each thickness of the phase-change material are shown;
2. At higher temperature, less VO_2_ thickness is needed to reach the minimum reflectivity compared to low temperature;
3. At longer wavelength, the minimum reflectivity occurs for less VO_2_ thickness than for a shorter wavelength;
4. The lowest reflectivity and highest GH shift occur at 630 nm and 95 °C, and, at a longer wavelength (785 nm), the lowest values occur at 20 °C.

Through performing a scan based on wavelength, the best configuration in terms of minimum reflectivity and GH shift, according to Table S6 in supporting information, is obtained when the sensor is excited at 735 nm, gold and VO_2_ thicknesses are equal to 49 and 1 nm, respectively, and the temperature of the phase-change material is fixed at 95 °C (1.048 × 10^−7^ minimum reflectivity and GH shift of 2.225 × 10^3^ μm). The best GH sensitivity, when Δn = 10^−10^ is attained, is when the sensor is excited at 770 nm, gold and VO_2_ thicknesses are equal to 46 and 4 nm, respectively, and the temperature of the phase-change material is fixed at 20 °C (1.393 × 10^8^ μm/RIU).

To further detail the influence of each parameter alone, two distinct studies are performed: first, the VO_2_ thickness is fixed, while that of the gold is tuned, and then vice versa. The results are then plotted and take the form of maximum GH shift and minimum reflectivity (Figures S2–S6 in supporting information).

### 3.2. Design with Metasurface

The GH shift can be further improved by replacing the continuous gold film with a metasurface based on a nanogroove configuration. The depth of the continuous film is chosen to be 30 nm, while that of the nanostructures is 20 nm (check Table S7 in supporting information). The width and periodicity of the grooves are the key variables of this setup and are optimized to yield the best results according to minimum reflectivity and GH shift.

Heating the phase-change material to 95 °C does not necessarily result in lower reflectivity and GH shift, even though the phase-change material has a higher absorption coefficient at higher temperature (Figure S8 in supporting information). Designing a metasurface structure with P = 140 nm, w = 20 nm, and VO_2_ thickness of 1 nm, which was heated at 95 °C and excited at 785 nm, yielded promising outcomes with a GH sensitivity in the order of 2.253 × 106 μm/RIU for Δn = 1.2 × 10^−6^, 1.738 × 10^6^ μm/RIU for Δn = 10^−10^ RIU, and a GH shift of 2.248 × 10^3^ (Table 1). Further improvements increased the GH sensitivity to 2.919 × 107 μm/RIU for Δn = 1.2 × 10^−6^ when λ = 1030 nm, 1 nm thickness of VO_2_, P = 130 nm, and w = 50 nm, with a FWHM of 1.095°; and GH shift equals 2.997 × 10^3^ μm when λ = 1000 nm, VO_2_ is 2 nm thickness, P = 110 nm, and w = 50 nm, with a FWHM of 1.454° (Table S8 in supporting information). The influences of both the grooves’ periodicity and width on the minimum reflectivity and thickness of VO_2_ are shown in the supporting information (Figures S8 and S9). Comparing the best configuration obtained in the simple continuous gold film case (thicknesses of 49 nm for gold and 1 nm for VO_2_, at 95 °C and an excitation of 735 nm) with that for a metasurface (continuous gold, metasurface, VO_2_ thicknesses of 30, 20, and 2 nm, respectively, at a temperature of 20 °C, excitation of 1000 nm, periodicity and width of 110 and 50 nm, respectively), an increase of 772 μm (from 2.225 × 10^3^ in the continuous gold configuration to 2.997 × 10^3^ μm in the metasurface configuration) in GH shift is shown.

**Figure 6:**
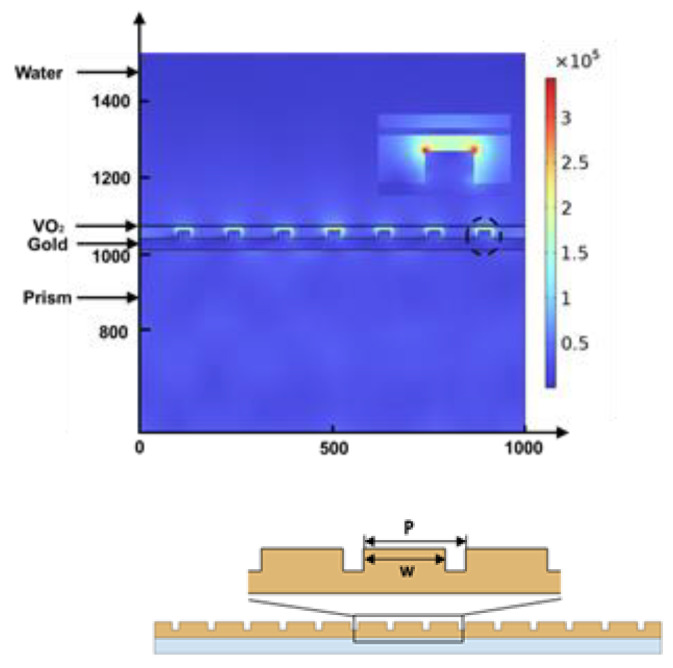
Electric field (norm in V/m) distribution inside the structure, given that the thickness of the continuous gold film, metasurface, and VO_2_ are 30, 20, and 4 nm, respectively, for excitation 630 nm, temperature 95 °C, periodicity 130 nm, and width 30 nm.

**Figure 7:**
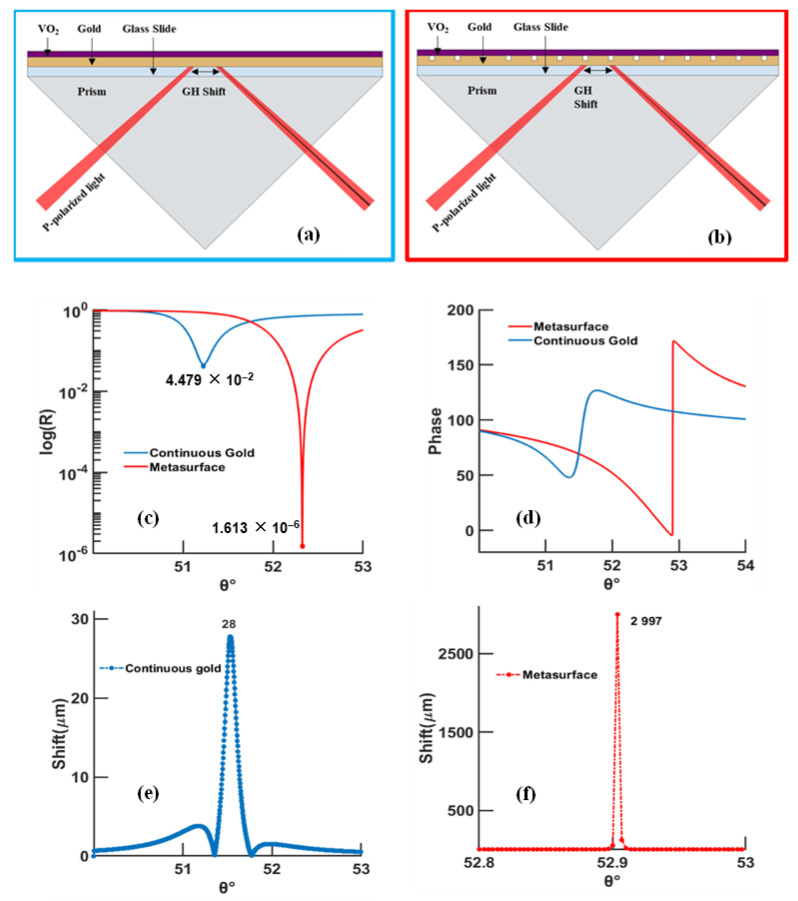
SPR curves calculated by TMM model, comparing the continuous gold configuration and the one with a metasurface, for an excitation of 1000 nm and VO_2_ at 20°. The continuous gold thickness is equal to 50 nm, while the metasurface case includes a continuous gold layer of 30 nm and nanostructures at 20 nm depth; (**a**) schematic of the continuous gold configuration, (**b**) schematic of the metasurface configuration, (**c**) reflectivity, (**d**) phase, (**e**) Goos–Hänchen shift of continuous gold film (absolute value), and (**f**) Goos–Hänchen shift of gold metasurface (absolute value).

One method to enhance the performance of the sensor is through the introduction of metasurfaces; these subwavelength structures are responsible for (a) modulating the wavevector of the surface plasmons, (b) confining the surface plasmon waves (Figure 6), and (c) enhancing the GH shift (Figure 7). Through the periodic patterning of the gold, a ‘double SPR’ effect is created (Figure 6): at the surface, between the glass substrate and the continuous gold layer by way of the excitation of SPR (surface plasmon resonance) waves, and at the tip at of the nanogrooves, through the excitation of LSPR (localized surface plasmon). Additionally, the effective refractive index, at the tip and just below the VO_2_ layer, is higher than that at the continuous gold film; this effect will confine the surface plasmon in their seats between the nanogrooves and VO_2_ and enhance the electric field at the sensing layer [13]. Accordingly, the metasurface was designed by tuning the periodicity and width of the nanogrooves.

## 4. Discussion

As the work function of gold, ~5.54 ev, is higher than that of VO_2_ [23] in its conducting state, 5.3 ev, the electron exchange takes place from the material with a lower work function (VO_2_) to the material with a higher work function (gold). Small reflectivity coefficients (in the order of ~10^−6^ and lower) are obtained when approximately all of the reflected energy is absorbed by the phase-change material. For the same gold thickness, heating at 95 °C requires less VO_2_ thickness to reach the optimal value of the minimum reflectivity for both wavelengths (Tables S2–S5 in supporting information); this is due to the smaller real component of the dielectric function at higher temperature, and, therefore, a smaller k vector is needed to couple the incident radiation (Equation S9 and Table S1 in supporting information). Lower reflectivity values are obtained at 95 °C for a 630 nm excitation compared to 20 °C, due to the higher absorption coefficient related to the imaginary component of the refractive index (Table S1 in supporting information). For a 785 nm excitation, better results (minimum reflectivity) are mostly obtained at 20 °C (Tables S4 and S5 in supporting information), even though the absorption coefficient is higher at 95 °C; in this case, the light will be mostly absorbed by the substrate, and less energy is transmitted to generate the surface plasmon waves. In general, for a high wavelength (above 700 nm), VO_2_ at 20 °C will result in higher GH shifts (Table S8 in supporting information). The process of tuning the sensors according to their variables relies predominantly on finding an equilibrium between the absorption and energy losses linked to the excitation wavelength and the thicknesses of both gold and VO_2_.

The sensitivity to variation of phase for Δn = 1.2 × 10^−6^ is shown to be more pronounced than the resonant angular sensitivity. In the latter case, deeper VO_2_ layers are required to promote a larger resonant angle variation, which will enlarge the FWHM and, therefore, decrease the detection efficiency, as the accurate point of minimum reflectivity cannot be found with precision. The increase in the FWHM is directly related to the thickness of VO_2_, due to the prominent energy losses found for deeper thicknesses. On the other hand, the phase sensitivity is linked to the value of minimum reflectivity itself. When the reflectivity is in the order of 10^−6^ and lower, a singular behavior of the phase is observed and, consequently, a higher phase sensitivity to refractive index variations, due to the significant electric field at the sensing layer. In simple terms, at the point of minimum reflectivity, a maximum transfer of incident energy to the surface plasmons is presented; this excitation leads to the enhancement of the electric field. Even if a minor increase in the FWHM occurs, the extensively large enhancement field created will eventually overcome this obstacle.

Even though better GH shifts are shown at a high temperature of VO_2_ (at certain wavelengths), some antibodies (IgG, IgA, IgE, IgM) have no heat tolerance and can become irreversibly denatured [24]. One way to overcome this problem is by decreasing the transition temperature of VO_2_ to some extent, to not deteriorate the antibodies. This can be done through several methods, by doping metal ions, niobium [25], molybdenum [26], tungsten [27], terbium [28], or chromium [29], into the VO_2_ structure, by annealing [30] or by stress introduction into the structure [31]. These techniques are shown to alter the optical properties of the phase-change material; for instance, the change in refractive index is affected by the percentage of the doping ions. However, at low concentrations of dopants and at visible frequencies, no significant change is recorded [25,26,27,28,29,32,33]. Doping 1% of tungsten and 0.4% of niobium can lower the transition temperature by 25 °C and 13 °C respectively [25]. Additionally, by changing the wavelength of excitation, high GH shifts and sensitivities can still be obtained at low temperatures (Figure S8).

## 5. Conclusions

A surface plasmon SPR sensor, based on a VO_2_ vanadium dioxide phase-change material as the sensing layer, has been systematically investigated based on several optimized parameters: gold and VO_2_ thicknesses, excitation wavelength, and the dielectric constants of the phase-change materials based on temperature tuning. All of these parameters are optimized to a near-zero minimum reflectivity at SPR resonance, leading to a giant lateral position GH shift and an ultra-high sensitivity of the sensing head. The GH shift using an optimized periodic metasurface was considerably enhanced when comparted to the continuous gold configuration (2.997 × 10^3^ μm). The enhancement effect induced was due to the coupling of surface plasmon resonance waves at the continuous gold surface and the localized SPR plasmonic effect introduced by the periodic structures. High GH sensitivities for both high (8.762 × 10^4^ μm/RIU for Δn = 0.02 RIU) and low (1.393 × 10^8^ μm/RIU for Δn = 10^−10^ RIU) refractive index changes are discussed. Additionally, when comparing the GH shift of VO_2_ with that of other well-known phase-change materials such as Sb_2_S_3_ [20] and GST [19] (order of a few tens of micrometer GH shifts), a remarkable increase in the GH shift is shown. The precise tuning of the thickness and optical absorption of the phase-change materials as well as the period and width of the gold meta-arrays allow for the achievement of a high GH shift and ultra-high sensitivity to low refractive index changes.

## Figures and Tables

**Table 1:** Summary of the best configurations, when the metasurface thickness is 20 nm on top of a 30 nm continuous gold film. The results are classified according to excitation wavelength, temperature, periodicity and width of the nanogrooves, thickness of VO_2_, minimum reflectivity, FWHM, GH shift, and GH sensitivities, for ∆n = 1.2 × 10^−6^ RIU and ∆n = 10^−10^ RIU.

λ (nm)	T (°C)	P (nm)	w (nm)	VO_2_ (nm)	Reflectivity	FWHM (°)	GH Shift (μm)	S_GH_ (μm/RIU) ∆n = 1.2 × 10^−6^	S_GH_ (μm/RIU) ∆n = 1 × 10^−10^
785	95	140	20	1	1.549 × 10^−6^	1.657	2.248 × 10^3^	2.253 × 10^6^	1.738 × 10^6^
785	20	130	20	2	3.542 × 10^−7^	1.954	1.359 × 10^3^	5.046 × 10^7^	4.885 × 10^7^

## Supplementary Materials

The following supporting information can be downloaded at: https://www.mdpi.com/article/10.3390/bios12100866/s1, Figure S1. SPR curves: minimum reflectivity versus wavelength and incident angle, VO_2_ at 20° with a thickness of 4 nm and a gold thickness of 46 nm; (a) reflectivity and (b) phase; Figure S2. Minimum reflectivity versus thickness of VO_2_ at 20 °C and 95 °C, for an excitation of 630 nm; Figure S3. Minimum reflectivity in function of both gold and VO_2_ thicknesses at 95 °C, for an ex-citation of 630 nm; Figure S4. Minimum reflectivity versus thickness of gold, when VO_2_ is at 20 °C and 95 °C and excited at 785 nm. Thickness of VO_2_ is (a) 2 nm, (b) 3 nm, (c) 4 nm, and (d) 5 nm; Figure S5. Width of the reflectivity curve versus gold thickness, when VO_2_ is at 20 °C with a 1 nm thickness, for excitations of (a) 630 nm and (b) 785 nm; Figure S6. Maximum Goos–Hänchen shift versus gold thickness, for a thickness of VO_2_ of 1 nm at 20 °C and 95 °C, for an excitation of (a) 630 nm and (b) 785 nm; Figure S7. Maximum GH shift versus the change in ∆n at 630 nm, with thicknesses of 47 nm and 2 nm of gold and VO_2_, respectively, and a temperature of 95 °C; Figure S8. Minimum reflectivity versus periodicity, when the nanogroove’s width equals 30 nm, with an excitation of 785 nm and VO_2_ at 95 °C, and (a) 1 nm and (b) 2 nm; Figure S9. Minimum reflectivity versus width, when the nanogroove’s periodicity equals 150 nm, with an excitation of 785 nm and VO_2_ at 95 °C, and (a) 1 nm, (b) 2 nm; Table S1. Refractive index of VO_2_ at 20 °C and 95 °C excited at 630 and 785 nm; Table S2. Summary of the best results of VO_2_ at 20 °C with an excitation of 630 nm, taking into consideration the gold and VO_2_ thicknesses, minimum reflectivity, FHWM, GH shift, GH sensi-tivity for ∆n = 0.02, angular sensitivity for ∆n = 1.2 × 10^−6^, phase sensitivity for ∆n = 1.2 × 10^−6^, and GH sensitivity for ∆n = 1.2 × 10^−6^; Table S3. Summary of the best results of VO_2_ at 95 °C with an excitation of 630 nm, taking into consideration the gold and VO_2_ thicknesses, minimum reflectivity, FHWM, GH shift, GH sensitivity for ∆n = 0.02, angular sensitivity for ∆n = 1.2 × 10^−6^, phase sensitivity for ∆n = 1.2 × 10^−6^, and GH sensitivity for ∆n = 1.2 × 10^−6^; Table S4. Summary of the best results of VO_2_ at 20 °C with an excitation of 785 nm, taking into consideration the gold and VO_2_ thicknesses, minimum reflectivity, FHWM, GH shift, GH sensitivity for ∆n = 0.02, angular sensitivity for ∆n = 1.2 × 10^−6^, and GH sensitivity for ∆n = 1.2 × 10^−6^; Table S5. Summary of the best results of VO_2_ at 95 °C with an excitation of 785 nm, taking into consideration the gold and VO_2_ thicknesses, minimum reflectivity, FHWM, GH shift, GH sensitivity for ∆n = 0.02, angular sensitivity for ∆n = 1.2 × 10^−6^, and GH sensitivity for ∆n = 1.2 × 10^−6^; Table S6. Summary of the best results of VO_2_, taking into consideration gold and VO_2_ thicknesses, excitation wavelength, temperature, minimum reflectivity, FHWM, GH shift, and GH sensitivity for ∆n = 0.02, ∆n = 1.2 × 10^−6^, and ∆n = 1.2 × 10^−10^; Table S7. Summary of the best results of VO_2_, while tuning the continuous and metasurface gold thickness, with an excitation of 630 nm, taking into consideration the temperature, periodicity, and width of the metasurface and thicknesses of continuous gold film, metasurface, and VO_2_, respectively, as well as minimum reflectivity, FHWM, GH shift, and GH sensitivity for ∆n = 0.02, ∆n = 1.2 × 10^−6^, and ∆n = 10^−10^; Table S8. Summary of the best results obtained when the continuous gold and metasuraface thicknesses are 30 nm and 20 nm, respectively, with VO_2_ optimized at 20 °C, taking into consideration the thickness of VO_2_ and excitation wavelength, periodicity, and width of the metasurface, as well as minimum reflectivity, FHWM, GH shift, GH sensitivity for ∆n = 0.02 and ∆n = 10^−10^, and angular sensitivity for ∆n = 1.2 × 10^−6^; Equations (S1)–(S20).
