# Supplementary material for: Highly Sensitive Plasmonic Biosensors with Precise Phase Singularity Coupling on the Metastructures
Source: Biosensors (Basel). 2022 Oct 12;12(10):866. doi: 10.3390/bios12100866 (PMC9599844; doi:10.3390/bios12100866)
Supplement: Supplementary file 1 [file biosensors-12-00866-s001.zip › biosensors-1924521-supplementary.pdf]

biosensors

Supporting Information:

Highly Sensitive Plasmonic Biosensors with Precise Phase Singularity Coupling on the Metastructures

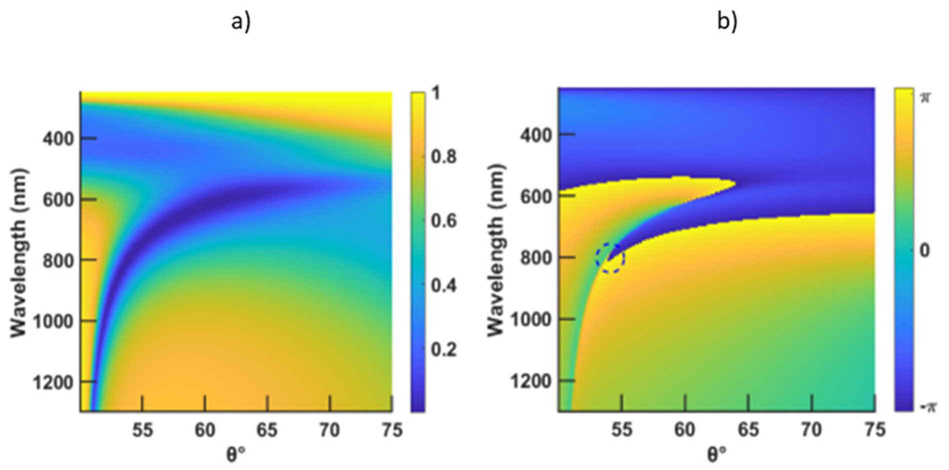

Figure S1. SPR curves: minimum reflectivity versus wavelength and incident angle, VO_2 at 20° with a thickness of 4 nm and a gold thickness of 46 nm; a) reflectivity and b) phase.

Table S1. Refractive index of VO_2 at 20 ℃ and 95 ℃ excited at 630 and 785 nm.

λ (nm)	20 °C	95 °C
630	3.159 + 0.473i	2.582 + 0.527i
785	3.022 + 0.489i	2.0625 + 0.748i

Table S2. Summary of the best results of VO_2 at 20 °C with an excitation of 630 nm, taking into consideration the gold and VO_2 thicknesses, minimum reflectivity, FHWM, GH shift, GH sensitivity for Δn = 0.02, angular sensitivity for Δn = 1.2 x 10^{-6}, phase sensitivity for Δn = 1.2 x 10^{-6}, and GH sensitivity for Δn = 1.2 x 10^{-6}.

Gold (nm)	VO_2 (nm)	Reflectivity	FWHM (°)	GH shift (µm)	S_{GH} (µm/RIU) Δn = 0.02	S_A (°/RIU) Δn = 1.2 x 10^{-6}	S_P (°/RIU) Δn = 1.2 x 10^{-6}	S_{GH} (µm/RIU) Δn = 1.2 x 10^{-6}
49	-	1.037 x 10^{-04}	2.808	94.02	3.418 x 10^3	0	1.183 x 10^{11}	9.736 x 10^3
46	4	2.608 x 10^{-05}	4.753	86.01	3.691 x 10^3	0	1.164 x 10^{11}	3.743 x 10^4
47	3	9.168 x 10^{-05}	4.208	54.08	1.432 x 10^3	0	7.269 x 10^{10}	2.391 x 10^3
48	1	8.922 x 10^{-05}	3.221	82.65	3.098 x 10^3	0	1.062 x 10^{11}	2.037 x 10^3

Table S3. Summary of the best results of VO_2 at 95 °C with an excitation of 630 nm, taking into consideration the gold and VO_2 thicknesses, minimum reflectivity, FHWM, GH shift, GH sensitivity for Δn = 0.02, angular sensitivity for Δn = 1.2 x 10^{-6}, phase sensitivity for Δn = 1.2 x 10^{-6}, and GH sensitivity for Δn = 1.2 x 10^{-6}.

**Gold (nm)**	**VO_2 (nm)**	**Reflectivity**	**FWHM (°)**	**GH shift (μm)**	**S_{GH} (μm/RIU) Δn = 0.02**	**S_A (°/RIU) Δn = 1.2 x 10^{-6}**	**S_P (°/RIU) Δn = 1.2 x 10^{-6}**	**S_{GH} (μm/RIU) Δn = 1.2 x 10^{-6}**
46	3	3.057 x 10^{-06}	3.771	2.637 x 10^{2}	1.524 x 10^{4}	74.000	8.150 x 10^{4}	7.872 x 10^{5}
47	2	1.281 x 10^{-08}	3.433	1.595 x 10^{3}	9.191 x 10^{4}	73.000	3.902 x 10^{4}	3.292 x 10^{7}
48	1	7.328 x 10^{-06}	3.111	2.911 x 10^{2}	1.322 x 10^{4}	72.765	1.451 x 10^{4}	2.305 x 10^{5}

Table S4. Summary of the best results of VO_2 at 20 °C with an excitation of 785 nm, taking into consideration the gold and VO_2 thicknesses, minimum reflectivity, FHWM, GH shift, GH sensitivity for Δn = 0.02, angular sensitivity for Δn = 1.2 x 10^{-6}, and GH sensitivity for Δn = 1.2 x 10^{-6}.

**Gold (nm)**	**VO_2 (nm)**	**Reflectivity**	**FWHM (°)**	**GH shift (μm)**	**S_{GH} (μm/RIU) Δn = 0.02**	**S_A (°/RIU) Δn = 1.2 x 10^{-6}**	**S_{GH} (μm/RIU) Δn = 1.2 x 10^{-6}**
50	-	1.351 x 10^{-3}	1.265	1.221 x 10^{2}	2.663 x 10^{3}	62.763	2.240 x 10^{5}
46	4	8.239 x 10^{-5}	2.053	1.788 x 10^{2}	5.922 x 10^{3}	67.764	2.158 x 10^{4}
47	3	2.572 x 10^{-5}	1.828	4.553 x 10^{2}	1.735 x 10^{4}	66.513	1.260 x 10^{6}
48	2	1.539 x 10^{-5}	1.623	7.891 x 10^{2}	3.463 x 10^{4}	65.263	3.218 x 10^{5}

Table S5. Summary of the best results of VO_2 at 95 °C with an excitation of 785 nm, taking into consideration the gold and VO_2 thicknesses, minimum reflectivity, FHWM, GH shift, GH sensitivity for Δn = 0.02, angular sensitivity for Δn = 1.2 x 10^{-6}, and GH sensitivity for Δn = 1.2 x 10^{-6}.

**Gold (nm)**	**VO_2 (nm)**	**Reflectivity**	**FWHM (°)**	**GH shift (μm)**	**S_{GH} (μm/RIU) Δn = 0.02**	**S_A (°/RIU) Δn = 1.2 x 10^{-6}**	**S_{GH} (μm/RIU) Δn = 1.2 x 10^{-6}**
46	2	4.481 x 10^{-5}	1.523	3.130 x 10^{2}	1.183 x 10^{4}	64.013	4.224 x 10^{5}
47	1	1.709 x 10^{-3}	1.398	4.444 x 10^{1}	1.692 x 10^{3}	63.513	8.369 x 10^{3}
48	1	4.113 x 10^{-5}	1.390	5.456 x 10^{2}	2.176 x 10^{4}	63.263	2.067 x 10^{6}

Table S6. Summary of the best results of VO_2, taking into consideration gold and VO_2 thicknesses, excitation wavelength, temperature, minimum reflectivity, FHWM, GH shift, and GH sensitivity for Δn = 0.02, Δn = 1.2 x 10^{-6}, and Δn = 1.2 x 10^{-10}.

**Gold (nm)**	**VO_2 (nm)**	**λ (nm)**	**T (°C)**	**Reflectivity**	**FWHM (°)**	**GH shift (μm)**	**S_{GH} (μm/RIU) Δn = 0.02**	**S_{GH} (°/RIU) Δn = 1.2 x 10^{-6}**	**S_{GH} (μm/RIU) Δn = 1 x 10^{-10}**
45	2	950	20	1.737 x 10^{-6}	0.923	1.677 x 10^{3}	8.284 x 10^{4}	4.849 x 10^{7}	5.084 x 10^{7}
46	4	770	20	1.773 x 10^{-8}	2.195	1.752 x 10^{3}	8.762 x 10^{4}	1.663 x 10^{8}	1.393 x 10^{8}
46	5	710	20	1.396 x 10^{-7}	3.333	1.966 x 10^{3}	8.953 x 10^{4}	7.263 x 10^{6}	8.025 x 10^{6}
47	1	935	20	4.934 x 10^{-6}	0.848	1.275 x 10^{3}	5.054 x 10^{4}	4.002 x 10^{7}	4.050 x 10^{7}
49	1	735	95	1.048 x 10^{-7}	1.735	2.225 x 10^{3}	1.020 x 10^{5}	1.668 x 10^{7}	1.917 x 10^{7}

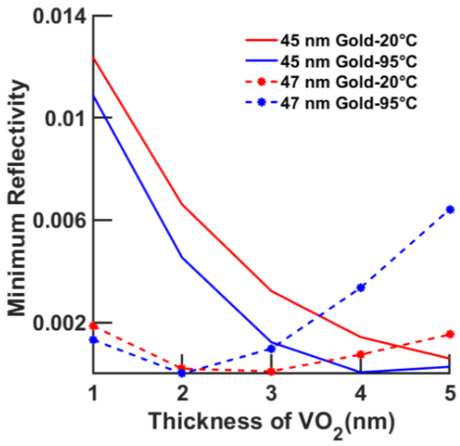

Figure S2. Minimum reflectivity versus thickness of VO_2 at 20 °C and 95 °C, for an excitation of 630 nm.

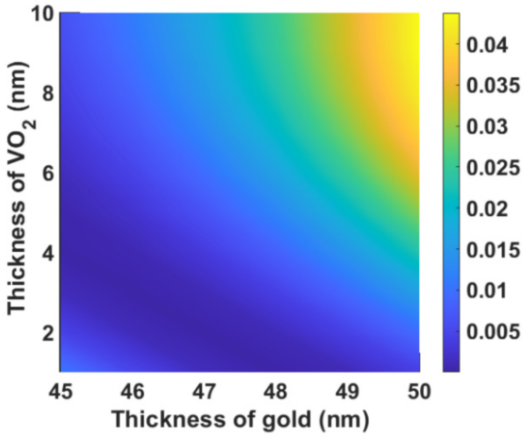

Figure S3. Minimum reflectivity in function of both gold and VO_2 thicknesses at 95 °C, for an excitation of 630 nm.

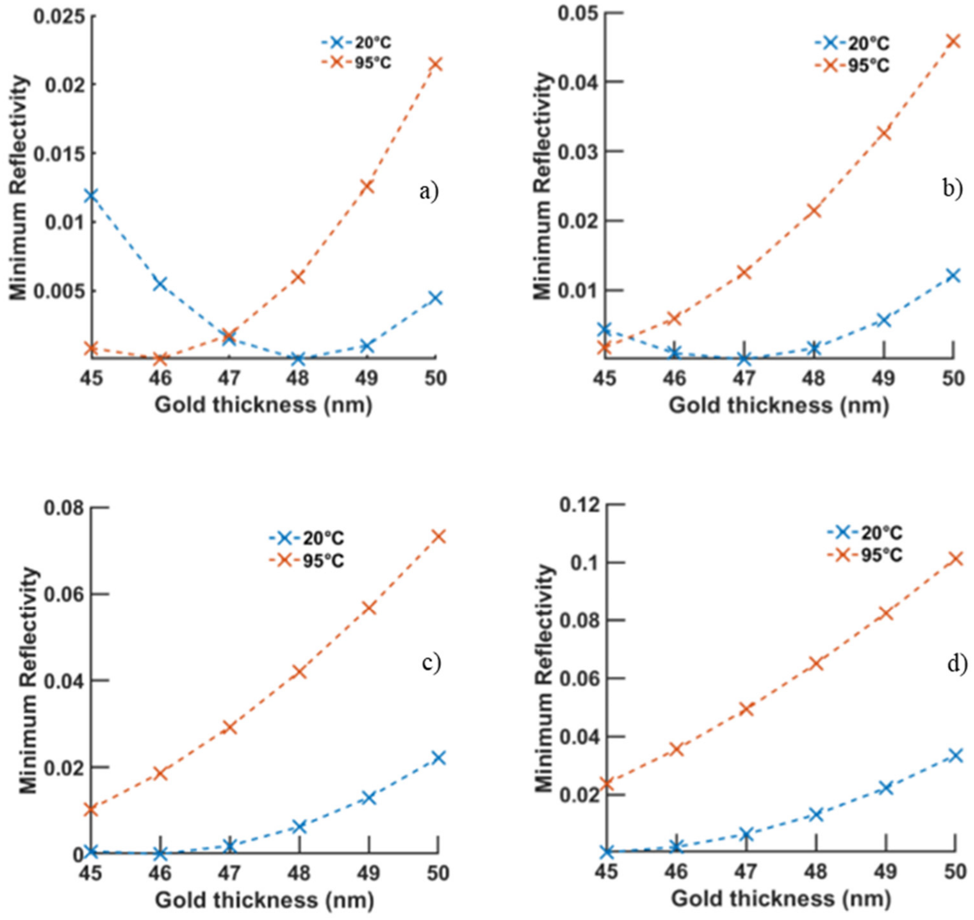

Figure S4. Minimum reflectivity versus thickness of gold, when VO_2 is at 20 °C and 95 °C and excited at 785 nm. Thickness of VO_2 is a) 2 nm, b) 3 nm, c) 4 nm, and d) 5 nm.

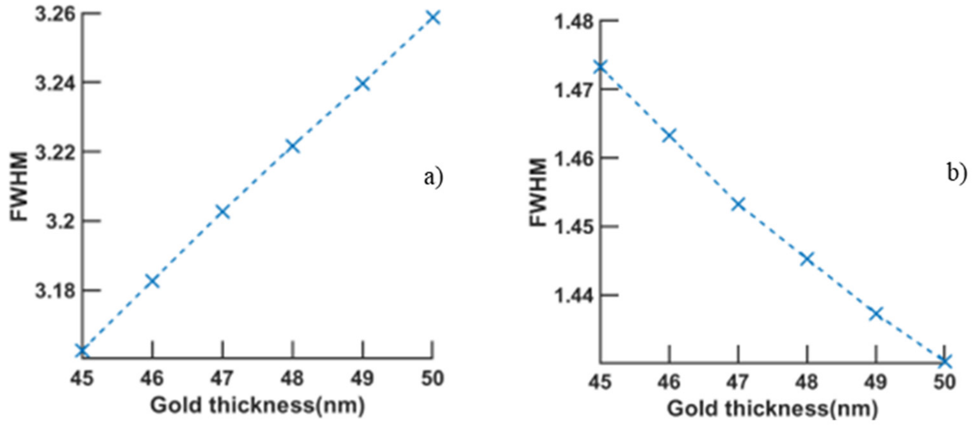

Figure S5. Width of the reflectivity curve versus gold thickness, when VO_2 is at 20 °C with a 1 nm thickness, for excitations of a) 630 nm and b) 785 nm.

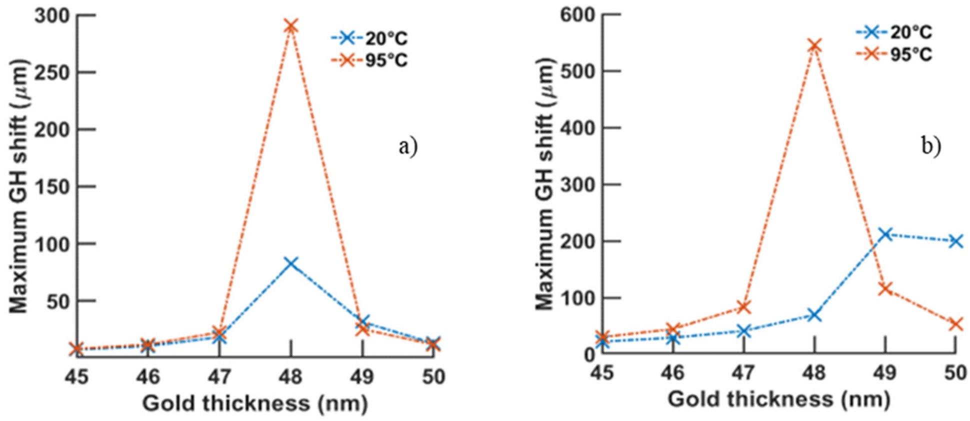

Figure S6. Maximum Goos–Hänchen shift versus gold thickness, for a thickness of VO_2 of 1 nm at 20 °C and 95 °C, for an excitation of a) 630 nm and b) 785 nm.

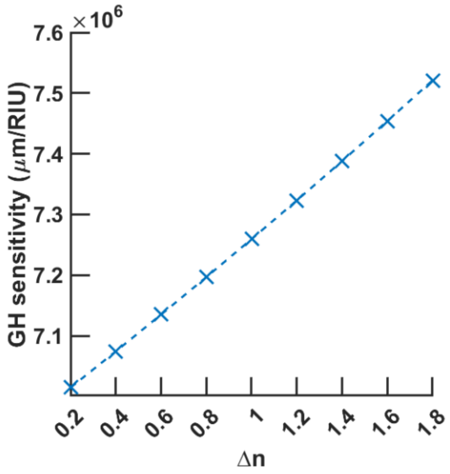

Figure S7. Maximum GH shift versus the change in Δn at 630 nm, with thicknesses of 47 nm and 2 nm of gold and VO_2, respectively, and a temperature of 95 °C.

Table S7. Summary of the best results of VO_2, while tuning the continuous and metasurface gold thickness, with an excitation of 630 nm, taking into consideration the temperature, periodicity, and width of the metasurface and thicknesses of continuous gold film, metasurface, and VO_2, respectively, as well as minimum reflectivity, FHWM, GH shift, and GH sensitivity for Δn = 0.02, Δn = 1.2 x 10^{-6}, and Δn = 10^{-10}.

T (°C)	P (nm)	w (nm)	Cont gold (nm)	Meta-surface (nm)	VO_2 (nm)	R	FWHM	GH Shift (mm)	S_{GH} (μm/RIU) Δn = 0.02	S_{GH} (μm/RIU) Δn = 1.2 x 10^{-6}	S_{GH} (μm/RIU) Δn = 10^{-10}
20	110	30	32	18	3	3.650 x 10^{-8}	5.856	1.347	6.680 x 10^{4}	1.471 x 10^{7}	1.357 x 10^{7}
20	150	20	28	22	1	3.544 x 10^{-7}	3.833	1.305	6.410 x 10^{4}	2.114 x 10^{6}	1.652 x 10^{6}
95	120	50	40	10	2	6.997 x 10^{-8}	5.014	2.008	9.960 x 10^{4}	5.102 x 10^{6}	4.996 x 10^{6}
95	140	50	28	22	4	2.003 x 10^{-8}	6.644	1.898	9.430 x 10^{4}	3.839 x 10^{6}	3.788 x 10^{6}
95	150	20	24	26	1	2.413 x 10^{-9}	3.736	1.911	9.430 x 10^{4}	8.541 x 10^{7}	5.385 x 10^{7}

Table S8. Summary of the best results obtained when the continuous gold and metasuraface thicknesses are 30 nm and 20 nm, respectively, with VO_2 optimized at 20 °C, taking into consideration the thickness of VO_2 and excitation wavelength, periodicity, and width of the metasurface, as well as minimum reflectivity, FHWM, GH shift, GH sensitivity for $\Delta n = 0.02$ and $\Delta n = 10^{-10}$, and angular sensitivity for $\Delta n = 1.2 \times 10^{-6}$.

VO_2 (nm)	λ (nm)	P (nm)	w (nm)	Reflectivity	FWHM (°)	GH shift (µm)	S_{GH} (µm/RIU) $\Delta n = 0.02$	S_A (°/RIU) $\Delta n = 1.2 \times 10^{-6}$	S_{GH} (µm/RIU) $\Delta n = 1 \times 10^{-10}$
1	980	130	40	5.197×10^{-6}	1.113	2.642×10^{3}	1.200×10^{5}	4.472×10^{6}	3.655×10^{6}
1	1030	130	50	1.553×10^{-6}	1.095	2.822×10^{3}	1.280×10^{5}	2.919×10^{7}	2.642×10^{7}
1	820	130	10	5.926×10^{-7}	1.367	2.595×10^{3}	1.240×10^{5}	4.954×10^{6}	5.442×10^{6}
1	995	120	40	2.764×10^{-6}	1.110	2.684×10^{3}	1.230×10^{5}	1.847×10^{7}	1.697×10^{7}
2	1000	110	50	1.613×10^{-6}	1.454	2.997×10^{3}	1.410×10^{5}	5.764×10^{6}	5.111×10^{6}

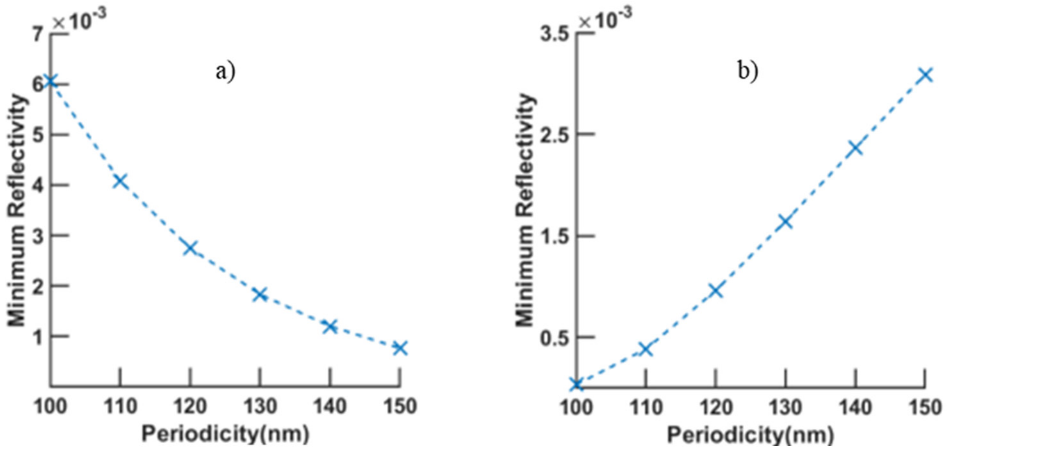

Figure S8. Minimum reflectivity versus periodicity, when the nanogroove's width equals 30 nm, with an excitation of 785 nm and VO_2 at 95 °C, and a) 1 nm and b) 2 nm.

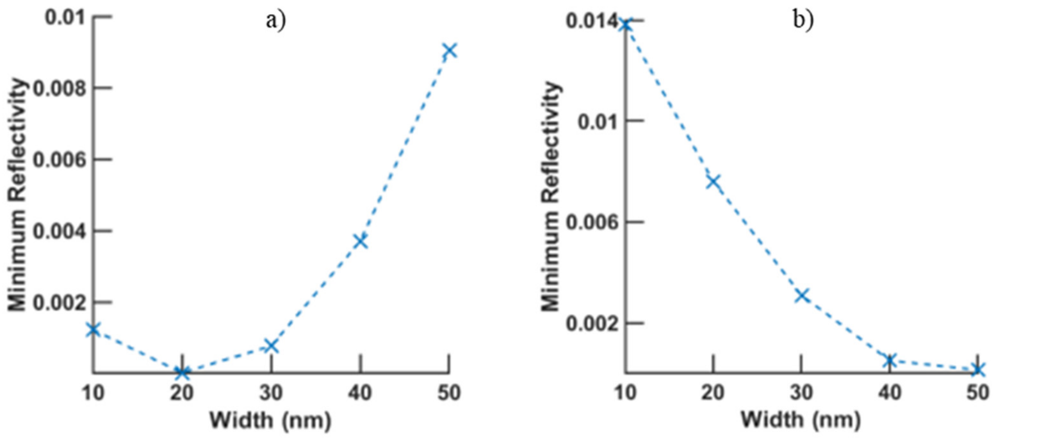

Figure S9. Minimum reflectivity versus width, when the nanogroove's periodicity equals 150 nm, with an excitation of 785 nm and VO_2 at 95 °C, and a) 1 nm, b) 2 nm.

Formatting of Mathematical Components

The SPR sensor follows the famous Kretschmann configuration; therefore, the coupling medium is an equilateral SF11 glass prism, and its refractive index is denoted as n_1. Its dispersion relation is given by the following equation:

$$n_1^2 - 1 = \frac{1.89878101\lambda^2}{\lambda^2 - 155.23629} + \frac{1.73759695\lambda^2}{\lambda^2 - 0.013188707} + \frac{0.313747346\lambda^2}{\lambda^2 - 0.0623068142} \quad \text{(S1)}$$

Where λ is the laser wavelength used for excitation, which is given in μm. The relation given in (S1) is applicable only when the wavelength is in the range of 0.37 to 2.5 μm. The following layer consists of a glass BK7 substrate with a thickness of 100 nm; its refractive index, n2, is determined by the following dispersion relation:

$$n_2^2 - 1 = \frac{1.039612122\lambda^2}{\lambda^2 - 0.00600069867} + \frac{1.01046945\lambda^2}{\lambda^2 - 103.560653} + \frac{0.231792344\lambda^2}{\lambda^2 - 0.0200179144} \quad \text{(S2)}$$

Again, λ is the laser wavelength used for excitation, which is given in μm. (S2 is operational when the wavelength is in the range of 0.3 to 2.5 μm, in which the transmittance is approximately 100%. The upcoming layer is the gold metal, and its refractive index, n_3, is taken from the Lorentz–Drude model. The final sensing layer is that of the change material, and its refractive index, n_4, is taken from experimental data.

To evaluate the efficiency of the sensor and quantify its performance, we used the well-known transfer matrix method (TMM) for a stratified medium, along with a Fresnel formulation.

$$M = \prod_{n=1}^{n=k-1} M_n = \begin{pmatrix} M_{11} & M_{12} \\ M_{21} & M_{22} \end{pmatrix} \quad \text{(S3)}$$

With:

$$M_{11} = M_{22} = \cos\beta_n \quad \text{(S4)}$$

$$M_{12} = -i\sin\beta_n / p_n \quad \text{(S4)}$$

$$M_{21} = p_n^2 M_{12} \quad \text{(S5)}$$

Where:

$$p_n = \frac{n_n^2 - n_1^2 \sin^2\theta_1}{n_n^2} \quad \text{(S6)}$$

$$\beta_n = p_n (k_1 d_n) n_n^2 \quad \text{(S7)}$$

Given that:

- N is the total number of layers, and n represents the index of the layer;
- θ_1 is the incidence angle (with respect to z) at the coupling medium, which is at the prism-glass substrate interface;
- k is the incidence wavevector in the coupling medium, which is the prism-glass substrate interface: $k_1 = \frac{2\pi}{\lambda_1}$.

The surface plasmon waves are only excited through the transverse magnetic mode (TM, p mode) when the incidence wavevector matches that of the plasmons, whereas the s or TE electric mode serves as a reference signal:

$$k_{SPP} = \mathrm{Re}\left[k_0 \left(\frac{\varepsilon_{PSM} n_s^2}{\varepsilon_{PSM} + n_s^2} \right)^{1/2} \right] \quad \text{(S8)}$$

where k_0 is the incident wavevector, in rad/m, ε_{PSM} is the permittivity of the phase change material, and n_s is the refractive index of the surrounding medium (water).

Given that, the reflection coefficient for the TM mode is set as:

$$\mathrm{r}_{TM} = \frac{(M_{11} + M_{12} p_n) p_1 - (M_{21} + M_{22} p_n)}{(M_{11} + M_{12} p_n) p_1 + (M_{21} + M_{22} p_n)} \quad \text{(S9)}$$

where the subscripts n and 1 denote the p coefficient for the first (prism) and nth layer, respectively. The reflectivity and phase are derived from

$$R_{TM} = |\mathrm{r}_{TM}|^2 \quad \text{(S10)}$$

$$\varphi_{TM} = angle(\mathrm{r}_{TM}) \quad \text{(S11))}$$

Accordingly, the GH shift is given as follows:

$$\mathrm{L}_{GH} = -\frac{1}{k_1 n_1} \frac{d\varphi}{d\theta} \quad \text{(S12)}$$

Along with minimum reflectivity and GH shift, FWHM and GH sensitivity are employed to evaluate the efficiency of the SPR sensor:

$$FWHM = \frac{1}{2}(\theta_{SPR} - \theta_{\min}) \quad \text{(S13)}$$

$$\mathrm{S}_{GH} = \frac{\Delta \mathrm{L}_{GH}}{\Delta n} \quad \text{S14)}$$

$$S_A = \frac{\Delta \theta}{\Delta n} \quad \text{(S15)}$$

$$S_P = \frac{\Delta \varphi}{\Delta n} \quad \text{(S16)}$$

where θ_{SPR} and θ_{min} are the angles of minimum reflectivity and minimum angle in the SPR curve, respectively, S_{GH}, S_A, and S_P are the GH, angular, and phase sensitivity for a Δn change in $\mu m/RIU$ and $°/RIU$, respectively.

The effective medium theory (EMT) is employed to design the metasurface, consisting of a periodic arrangement of nanogrooves of width w and separated by P, which denotes the periodicity. The formulation used is known as the volume fraction approximation and can be employed for tuning the model by estimating the relative permittivity of a composite mixture. It shows accurate results when parameters P and w are in the deep subwavelength regime (P and w < λ/10).

$$\varepsilon_{//} = f\varepsilon_{gold} + (1-f)\varepsilon_d \quad \text{(S17)}$$

$$\varepsilon_{\perp} = \frac{(1+f)\varepsilon_{gold}\varepsilon_d + (1-f)\varepsilon_d^2}{(1+f)\varepsilon_d + (1-f)\varepsilon_{gold}} \quad \text{(S18)}$$

where f is the volume filling ratio that describes the percentage of occupation of each element of the composite material:

$$f = \frac{P-w}{P} \quad \text{S20)}$$

$\varepsilon_{//}$ and $\varepsilon_{\perp}$ are the relative permittivities, parallel and perpendicular to the nanogrooves, respectively. In our simulation, we only used $\varepsilon_{//}$, as it has the greater influence in exciting the surface plasmons (parallel to the direction of free electron motion).
